# Mate Value and Self-Esteem: Evidence from Eight Cultural Groups

**DOI:** 10.1371/journal.pone.0036106

**Published:** 2012-04-25

**Authors:** Robin Goodwin, Tara Marshall, Marta Fülöp, Joseph Adonu, Slawomir Spiewak, Felix Neto, Sonia Hernandez Plaza

**Affiliations:** 1 Department of Psychology, Brunel University, London, United Kingdom; 2 Institute for Psychology, Hungarian Academy of Sciences, Budapest, Hungary; 3 Department of Psychology, University of Bedfordshire, Luton, United Kingdom; 4 Institute of Psychology, Jagiellonian University, Krakow, Poland; 5 Faculdade de Psicologia e de Ciências da Educação, University of Porto, Porto, Portugal; 6 Centre for Research and Studies in Sociology, University Institute of Lisbon (ISCTE-IUL), Lisbon University Institute, Lisbon, Portugal; Queen Mary, University of London, United Kingdom

## Abstract

This paper explores self-perceived mate value (SPMV), and its association with self-esteem, in eight cultures. 1066 participants, from 8 cultural groups in 7 countries, rated themselves on 24 SPMVs and completed a measure of self-esteem. Consistent with evolutionary theory, women were more likely to emphasise their caring and passionate romantic nature. In line with previous cross-cultural research, characteristics indicating passion and romance and social attractiveness were stressed more by respondents from individualistic cultures, and those higher on self-expression (rather than survival) values; characteristics indicative of maturity and confidence were more likely to be mentioned by those from Traditional, rather than Secular, cultures. Contrary to gender role theory, societal equality had only limited interactions with sex and SPMV, with honesty of greater significance for male self-esteem in societies with unequal gender roles. These results point to the importance of cultural and environmental factors in influencing self-perceived mate qualities, and are discussed in relation to broader debates about the impact of gender role equality on sex differences in personality and mating strategies.

## Introduction

A great deal of previous research has examined the partners we seek for a romantic relationship [1]. However, increasing attention has focused on the characteristics we believe we can offer a relationship partner, often termed “self-perceived mate value" (SPMV). SPMV is “one's assessment of one's own mate value (attractiveness) as compared to potential competitors" [2]. Such an estimate reflects our evaluation of our “bargaining power" in the relationships marketplace [3]. In doing so, it allows us to avoid wasting resources on aiming for mates we cannot achieve, or on less valuable mates who compromise our ability to produce viable offspring [4], [5].

Work on perceived mate values has been influenced by two theoretical traditions. From an evolutionary perspective, each species has a genetically organized set of strategies and tactics for survival, growth and reproduction [6]. Traits that maximize gene replication are considered fit and assumed to be targets of mate choice [7]. Research from this perspective suggests that, as men prefer women with characteristics that indicate their ability to produce viable offspring, women should value their youth, physical attractiveness and health when indicating their SPMV [1]. Women themselves prefer men with social status or dominance, indicators of resource potential that suggest men with these qualities can ‘provide’ for their family [8], [9]. This implies that characteristics such as earning potential, ambition and industriousness should be valued by men as important for attracting a potential partner. In addition, various environmental factors may influence the qualities that individuals feel are important for attracting a mate. Pathogen prevalence, and the resources available, may cause individuals to adjust their mating strategy to maximise their chances of successful reproduction, and consequently alter the qualities they value in a partner [10], [11]. According to modified parental investment theory (the BSD model) [12] stressful ecologies (often economically poorer societies, with harsher environmental conditions) encourage both men and women towards short term mating strategies, with low emotional investment and shorter term mating strategies. In contrast, lower cultural stress has been associated with stronger relationship parent-child attachment, which has contributed to greater subsequent emotional investment in close relationships [13].

A second theoretical perspective, social structural theory [14] also recognizes that partner preferences reflect adjustment to the environmental situation, but emphasizes the role of cultural divisions of labour in guiding self-perceived mate preferences [14], [15]. From this viewpoint, mate preferences reflect the maximization of outcomes for men and women within specific societies [15]. From this perspective, earning differentials in many societies mean that women prefer men with financial status, while men favour women who are nurturant ‘home makers’ [14]. Where large divisions of labour persist and gender inequality persists, we anticipate that men and women would also value such characteristics in themselves, as indicators of qualities attractive to a mate.

From both theoretical perspectives, perceived qualities have significant implications for self-esteem. According to sociometer theory [16] self-esteem represents an indicator of our exclusion from important relationships. Unsurprisingly, therefore, previous research has found positive correlations between overall SPMV and global self-esteem [10], [17]. Those who believe they possess few qualities valued by the market place feel less confident about their abilities of finding a marital partner [18]. In contrast, research based primarily in the US has suggested that those with high self-esteem are likely to believe they are intelligent, attractive and popular [19], while successful mating increases SPMV [20]. Importantly for the present paper, those who posses qualities matching the most desired sex-typed characteristics have been shown to possess the greatest esteem [21], [22].

At present, the great majority of the research on SPMV, and SPMV and self-esteem, has been conducted in the U.S. However, as noted above, both social structural theorists and evolutionary theorists recognize that social and ecological conditions influence the choice and evaluation of partners [21]. In our study we gathered data from White and British Indian, Ghanaian, Portugese, Polish, Chinese, Hungarian and Spanish respondents. We categorise these using the predominant categorizations of culture commonly used in the cross-cultural literature to formulate hypotheses about cultural variations in attitudes and behaviours [23]. Our first dimension is that of individualism-collectivism. While self-enhancement may be universal, previous research indicates that individuals from collectivist cultures self-enhance on collectivistic attributes, individualists on individualistic attributes [24]. Individualism-collectivism distinguishes between self-orientated, loosely connected (individualist) societies, where personal goals are primary, and more strongly integrated collectivist societies, where group solidarity is strong and where marital relationships link families, rather than just individuals [25]. As such, we anticipate group relations to be of greater significance in collectivist societies. Individualism and Schwartz's measures of autonomy are significantly correlated across a number of analyses [26], [27] Because of the lack of complete contemporary data on individualism and collectivism we use Schwartz's data on the related dimensions of Embeddedness and Hierarchy vs. Intellectual and Affective Autonomy [26], alongside Hofstede's data and score estimates for countries not in his original data set [25]. Ghana and China are placed within the ‘embedded’ ‘collectivist’ group, alongside British Asian respondents, who predominantly originate from India or Pakistan. European respondents (White British, Spanish, Portuguese, Hungarian and Poles) form the ‘individualist’ grouping.

We complement this with a second set of cultural dimensions - Survival values vs. Self-Expression values and Traditional vs. Secular values - from the World Values Survey [28]. Resource scarcity has been related to high earning potential, commitment and nurturing [29]. In societies where Survival values are important priority is given to economic and physical security [30]. SPMVs should reflect such priorities. In contrast, self-expressive cultures should value quality of life, emotionality and self-expression, with a desire for a wider range of personality characteristics (e.g. sociability and humour). Societies high on Traditional values emphasize traditional religions, strong parent-child ties, and deference to authority. Because conformity and family ties are highly valued emphasis in traditional cultures, there should be a more positive evaluation of characteristics suggesting a more mature, family orientated partner, rather than a focus on the individual relationship emotional concerns more valued in a secular rational culture [15], [31]–[33]. Using the World Value Survey value map [28] we place our Ghanaian, Chinese, British Asian, Hungarian and Polish respondents higher on the Survival dimension, White British, Portuguese and Spanish respondents higher on the Self Expression dimension. Ghanaian, British Asian, Portuguese and Polish respondents are located within the Tradition quadrant; Hungarian, Spanish, White British and Chinese participants within the Secular-Rational grouping. Finally, gender differentiation interacts with culture to influence SPMVs. Partner preferences are most differentiated between the sexes in societies where occupational behavior emphasizes differentiation [14]. We predict that in the more gender-differentiated societies women will favour men with financial status, while men favour women who are nurturant ‘home makers’.

### Overview of this paper

Previous work on SPMV has focused on research conducted in Western cultures, and has failed to explore cross-cultural variations in these values. In addition, most research has focused on the exchange of female beauty and male resources, but SPMV is a multi-faced concept that includes more than women's beauty or male resources [20]. In this study we examine a range of perceived mate value characteristics, cultural differences in these stated qualities, and the relationship between SMPVs and self-esteem. We suggest that holding positive relationship attributes is likely to be predictive of high self-esteem, but that self-enhancement is likely to be culture specific, with the correlation between particular SPMVs and self-esteem partly dependent on cultural group. Finally, we expect the impact of gender on the relationship between SPMVs and self-esteem to be moderated by culture, with particular gender differences in the SPMV-esteem relationship in the most gender-differentiated cultures.

## Methods

### Participants

Participants were 1066 students (59% female, *M* age 24.0, *SD* = 7.7) from eight different cultural groups. Two samples were collected in Britain, from British White and British Asian participants in the same London University. Further data were collected in major urban Universities in Ghana, Portugal, Poland, China, Hungary and Spain. All participants were recruited on the campuses during class time or following classes. Table 1 gives a breakdown of participants by cultural group and sex. This study was performed in strict accordance with the recommendations of the British Psychological Society. Ethical approval was given by the Ethics Committee of the Department of Psychology, Brunel University. All participants gave informed oral consent to their participation; as written consent is not the normal practice in several cultures in our study and is likely to be counterproductive in undermining confidence in the anonymity of procedures, we did not collect written consent forms. Respondents were given the opportunity to refuse to participate, to omit questions or withdraw from the study at any time without penalisation.

**Table 1:** Respondents, scale reliabilities, and Gender Equality Scores by Country.

Culture	N	Females (%)	Self-esteem α	SPMV α	Rank GDI (GEM score)	IND Score	TRADRAT Score	SURVIVALSELF Score
UK White	173	105 (62)	.86	.78	10 (0.78)	89	0.06	1.68
UK Asian	127	70 (56)	.66	.66	128/136 (0.37)^a^	48/14	−0.36/−1.42	−0.21/−1.25
Ghana	84	35 (42)	.79	.89	116 (no score)	15	−1.94	−0.29
China	109	50 (46)	.79	.92	72 (0.53)	20	0.80	−1.16
Portugal	198	100 (51)	.79	.88	28 (0.69)	27	−0.90	0.49
Poland	120	93 (80)	.86	.82	35 (0.61)	60	−0.78	−0.14
Hungary	170	113 (67)	.82	.72	34 (0.57)	80	0.40	−1.22
Spain	85	62 (73)	.82	.78	12 (0.79)	51	0.09	0.54
Totals	1066	628 (59)	.83	.85				

GDI Scores are for India and Pakistan respectively; most British Asians have family roots in these countries. In the GEM comparative database there was a score for Pakistan but not for India, so only the Pakistan figure is included. GDI/GEM scores are from the United Nations Development Report 2007 (http://hdr.undp.org/en/media/HDR_20072008_GEM.pdf, Accessed 2011 Nov 23). Individualism (IND) scores are from http://geert-hofstede.com/countries.html, Accessed 2012 Mar 26). The World Value Survey (WVS) Traditional vs Rational (TRADRAT) and Survival vs Self-Expression (SURVIVALSELF) scores are from http://www.worldvaluessurvey.org/wvs/articles/folder_published/article_base_111, Accessed 2012 Mar 26). WVS scores are from the latest survey waves in their respective countries (2000 or 2006).

### Measures

Pilot work at a London University generated 24 commonly occurring qualities that thought they possessed that a romantic partner might find attractive. The 24 item qualities obtained were then used to create an ‘SPMV index’. Participants were asked the extent to which they possessed each of the 24 characteristics (5 point scale, ranging from *not at all* to *a great deal*). Self-esteem was assessed using the 10-item Rosenberg Self-Esteem Scale, the most widely used and validated self-report measure of global self-esteem [34]. This scale has also been regularly used in previous studies of SPMV [17], [21]. Questionnaires were back-translated in each country where necessary, and given to participants in the local language.

Gender equality across cultures was measured using two measures included in earlier cross-cultural analyses of gender equality and partner preferences [15]; the Gender Development Index (GDI) and the Gender Empowerment Measure (GEM). The GDI indicates relative ranking of nations on a mixture of economic, education and health indicators, with a high rank (i.e. low number) indicating that women perform better on these indicators. The GEM examines focuses more on participation, and includes relative percentage of parliamentary seats held by women, women in key economic making decisions (e.g. managerial positions) and relative female share of income (compared to males). A high score indicates greater opportunities for women [15]. Table 1 provides country scores on gender equality.

## Results

Principal components analysis (with varimax rotation) reduced the 24 SPMV characteristics to five factors, which explained together 45% of the variance: *caring* (caring, good listener, supportive, honest, faithful, good worker: 12% variance); *socially attractive* (sociable, attractive, humorous, intelligent, stimulating, cultured: 11% variance); *passionate romantic* (passion, romance: 7% variance) *adventurer* (adventurer, athletically fit, independent, easygoing: 8% variance) and *mature confident* (mature, realistic, confident, generous with time and money, good cook: 7% variance).

We first considered the impact of culture and sex on the five SPMV factors. To meaningfully examine these cultural effects we employed the three cultural dimensions described above (Individualism/Collectivism, Secular/Traditional and Security/Self Expression values). Because these cultural dimensions overlap conceptually, we conducted three separate MANOVAs, with the cultural dimensions and sex as the independent variables and the five SPMV factors as criterion variables. Below we report the significant effects. In our first analysis, with Individualism/Collectivism as the cultural grouping, there were sex effects for the SPMV dimensions of *socially attractive* (*F* (1, 976) = 4.22, *p*<.04; with men higher on this dimension) and *passionate romantic* (*F* (1, 976) = 12.99, *p*<.001, with women higher on this dimension). There were also unique culture grouping effects for the SPMV dimensions of *caring* (*F* (1, 976) = 54.97, *p*<.001), *socially attractive* (*F* (1, 976) = 102.05, *p*<.001), *mature confident* (*F* (1, 976) = 26.46, *p*<.001), and *passionate romantic* (*F* (1, 976) = 63.77, *p*<.001). Participants from individualistic cultures were higher on *passionate romantic* (*M* = 7.79 (individualist) vs. 6.84 (collectivist)), *caring* (*M* = 24.73 (individualist) vs. 22.89 (collectivist culture)), *socially attractive* (*M* = 21.73 (individualist) vs 19.22 (collectivist culture)) and *mature confident* (*M* = 21.17 (individualist) vs. 19.84 (collectivist)). In addition, there was a sex x cultural grouping interaction on the *mature confident* dimension, with this dimension score highest amongst males from individualist cultures (*M* = 21.47), and lowest for males from collectivist cultures (*M* = 19.38)

We then turned to the Tradition-Secular dimension and Security-Self Expression cultures. Here there were unique sex effects for the *caring* dimension (*F* (1, 976), *F* = 5.26, p<.02) and for *passionate romantic* scores (*F* (1, 976) = 19.95, p<.001), with females higher on *caring* (*M* = 24.41 (women) vs. 23.88 (men))) and *passionate romantic* (*M* 7.73 (women), 7.23 (men)). There were unique culture effects for *mature confident* (*F* 1, 976) = 32.37, *p*<.001) and *passionate romantic* (*F* 1, 976) = 10.21 *p*<.001). In both cases traditional cultures scored higher on these dimensions (for *mature confident M* = 21.38 (traditional) vs. 20.03 (secular culture); for *passionate romantic* (*M = 7*. 66 (traditional) vs. 7.30 (secular)). There were also cultural grouping x sex effects for *caring* (*F* 1, 976) = 14.71 *p*<.001), *socially attractive* (*F* 1, 976) = 5.36 *p*<.02), *mature confident* (*F* 1, 976) = 9.40 *p*<.002) and *passionate romantic* (*F* 1, 976) = 20.19, *p*<.001). Highest on *caring* were women from secular cultures (*M* = 24.75), lowest on this dimension were men from secular cultures (*M* = 23.32). Highest on *socially attractive* were men from traditional culture (*M* 21.35), lowest were men from secular cultures (*M* = 20.68). Men from traditional cultures scored highest on *mature confident* (*M* = 21.70), men from secular cultures lowest on this dimension (*M* = 19.63). *Passionate romantic* was highest amongst females from secular cultures (*M* = 7.80), lowest for males from these cultures (*M* = 6.80).

For our final dimension, security-self expression, there were unique sex effects, with women higher on *caring* (*F* (1, 976) = 5.25 *p*<.02, *M* = 24.55 (women) vs. 24.02 (men)), and *passionate romantic* (*F* (1, 976) = 17.93, *p*<.001, *M* = 7.77 (women) vs. 7.30 (men)). There were unique culture effects on *caring* (*F* (1, 976) = 68.92, *p*<.001), *socially attractive* (*F* (1, 976) = 10.17, *p*<.001), *mature confident* (*F* (1, 976) = 101. 91, *p*<.001) and *passionate romantic* (*F* (1, 976) = 48.55, *p*<.001). Self expressive cultures scored higher on *caring* (*M*s = 25.24 (self expressive) vs. 23.33 (survival culture)), *socially attractive* (respective *M*s = 21.40 vs. 20.63), *mature confident* (respective *M*s = 22.03 vs 19.69) and *passionate romantic* (respective *M*s = 7.92 vs. 7.15). There were no sex x culture interactions.

Turning to the relationship between the SPMV factors and self-esteem, all eight cultural samples demonstrated a significant moderate correlation between total SPMV scores and self-esteem (ranging from .23 (White British respondents) to .49 (Polish sample), average sample *r* = .38). We conducted three multiple regressions, one for each cultural dimension (Individualism/Collectivism, Secular/Traditional and Security/Self Expression values), with self-esteem as the criterion variable. Predictors were the five centered SPMV factors, cultural grouping (e.g. Individualism vs. Collectivism) and the interaction between SPMV dimension and cultural group. Below we report the significant effects. *Socially attractive*, *mature confident*, and *passion romantic* all significantly correlated with self-esteem (all significant at *p*<.01, exact coefficients vary with other variables in the equation). There was no unique effect for Individualism/Collectivism on self esteem but an interaction between SPMV and cultural group, with *mature confident* more highly correlated with self-esteem for respondents from individualistic, versus collectivist, cultures (ß = −.11, *t* = −2.64, *p*<. 01). Those in Secular cultures enjoyed a greater degree of self-esteem (ß = −.17 t = −5.96, *p*<. 001; respective *Ms* = 3.80 (secular) vs. 3.60 (traditional) cultures), with *caring* more strongly correlated with self-esteem in traditional cultures (ß = .10 t = 2.84, *p*<. 01, respective *r*s .34 vs .16). Comparing survival and self-expressive cultures, there was a unique effect for cultural grouping on self-esteem (ß = .26 t = 8.38, *p*<. 001), with those in self-expressive cultures enjoying higher self-esteem (*M*s = 3.95 (expressive) vs. 3.52 (survival cultures)). Those who scored more highly on *caring* in survival–oriented cultures also scored higher on self-esteem (ß = .−08 t = −2.31, *p*<. 02; respective *r*s .27 vs .07).

Finally, we predicted a sex x cultural sex role egalitarianism interaction in the relationship between SPMV and self esteem. We created two new dummy variables, the first contrasting the more sex role egalitarian cultural groups (British White, Spain; average world ranking of 11 on the GDI) with the most unequal cultures (Ghana, British Asian; estimated world ranking 124); the second contrasting the gender equal and the other ‘medium’ scores on GDI (Portugal, Poland and Hungary and China; mean world ranking 42). We ran a multiple regression with self-esteem as the criterion variable and with the five SPMV factors as predictors, alongside sex, the dummy codes for sex role grouping, sex x sex role grouping, each SPMV x sex, each SPMV by sex role group, and the three way interactions for each SPMV by sex by sex role group. Men scored higher on self-esteem overall (ß = −.09, t = −2.62, *p*<. 01) and there was a unique effect for gender role differentiation, with the more equal cultures higher on self-esteem than the most unequal cultures (respective *Ms* of 3.91 vs. 3.60, ß = −.11, t = −2.80, *p*<. 01). There was a two way interaction for SPMV x sex for the *caring* factor (ß = −.09, t = −2.17, *p*<. 03), with men more strongly correlating *caring* with their self-esteem. However, a three-way interaction between *caring* x sex x sex role grouping also demonstrated greater sex differences in the relationships between esteem and this attribute in the more sex role unequal cultures (ß = −.09, t = −2.13, *p*<. 03). Hence while the correlation between self-esteem and *caring* was small for both men and women in the more sex-role equal societies (*r* (99) = .03 for men; *r* (164) = .07 for women), men were more likely to correlate esteem and *caring* in the more unequal sex-role cultures (*r* (99) = .49 and *r* (102) = .16 for men and women, respectively). A further analysis of the *caring* factor demonstrated that it is on the single SPMV attribute ‘honesty’ that the sex/culture groups most clearly differ. In the unequal sex-role societies it was men who most clearly related this attribute to their self-esteem (*r* (103) = .34 for men, *r* (104) = .05 for women, in the most unequal societies). In contrast, honesty was not closely correlated with self-esteem for either sex in the more equal gender-role societies (*r* (85) = −.04 for men, *r* (182) = −.10 for women). Finally, there was a three-way interaction between sex, the *adventurer* category of SPMV, and the contrast between gender egalitarian and ‘moderately’ gender equal cultures. In moderately egalitarian cultures, women's score on *adventurer* was more strongly correlated with their self-esteem (*r* (231) = .17 for men, *r* (354) = .33 for women).

## Discussion

In this study we examined differences in self-perceived mate value (SPMV) across eight cultural groups in seven countries, and the relationship between perceived mate value and self-esteem by gender and culture. When asked to rate key attributes for attracting a mate, women were more likely to emphasise their *caring* and *passionate romantic* nature. Ratings of attributes also varied by culture, and there were additional interactions between culture and sex. As in previous work, SPMV was a significant correlate of self-esteem. However, some sex differences in the relationship between valued traits and self-esteem were moderated by degree of sex-role equality in a society.

First, let us consider sex differences in the attributes emphasized by our participants. As predicted, women were keen to emphasise their *caring* and *passionate romantic* side in their self-ratings of attractiveness factors. These finding are largely consistent with evolutionary theories, which emphasise women's greater caring role and emotional investment in relationships [33], [35]. Further, post-hoc analysis of our *caring* dimension demonstrated that it was a self-perception of faithfulness which was more important for male self esteem (respective *rs* of .24 (men), .09 (women)), while supportiveness was more significant for female esteem (respective *r*s .22. and .11 (men, women)). There was no consistent significant sex difference in self-ratings of the broader factor of *socially attractive*, which consisted of both appearance but also a range of personality qualities. This failure to find sex differences in self-perceived attractiveness has been reported elsewhere [5], [22], and may reflect the increasing convergence in relationship attributes worldwide. Indeed, a post-hoc comparison showed no significant sex differences on the individual attributes that comprised this factor. Consistent with previous work [36] men in our sample also scored higher on self-esteem.

As discussed above, both social role and evolutionary perspectives recognize that there can be important cultural variations in the qualities valued in a partner. As anticipated, participants in individualist societies were more likely to highly rate qualities associated with emotional investment in a relationship [33]. In contrast, in collectivist societies, romance and passion may challenge family authority [1], with parents or kin instead choosing mate partners for their offspring on the basis of their economic and family background [37]. It was therefore not surprising to find that the SPMV factors of *passionate romantic* and *socially attractive* (e.g. sociable, attractive, humorous) were less stressed by respondents from collectivist cultures. *Mature confident* was also more likely to be mentioned by those from Traditional, rather than Secular, cultures, as hypothesized. The BSD evolutionary model [12] suggests that economic pressures may significantly impact on partner choice, an observation also made by several cultural and political theorists [38], [39]. Consistent with our expectations, in cultures where survival pressures were less important (i.e. those higher on self-expression), *socially attractive* and *passionate romantic* was more frequently mentioned as attractive characteristic possessed by our respondents. While our participants were more likely to describe themselves as *caring* in individualist and self-expressive societies, *caring* was positively correlated with self-esteem in the more traditional and security-orientated cultures. Thus while being humorous, cultured or sociable may be particularly important in richer societies, where survival obligations are less prominent, being able to care for others may be important for esteem where economic survival is threatened.

From a social role perspective, we would expect role equalities in the division of labour to influence the qualities emphasized by individuals as attractive to mates, and the relationship between these qualities and self-esteem. Here we received only mixed support for the impact of societal gender-role differentiation on the relationship between self-perceived mate attributes and esteem. Contrary to our expectations, there was only one interaction effect between perceived attributes, societal equality and sex, with men who scored more highly on *caring* scoring higher on self-esteem in less egalitarian cultures. This finding resulted primarily from differences in the single attribute of ‘honesty’, with honesty of greater significance to men in sex unequal societies than elsewhere. Given that the sexual infidelity of men may of particular issue in cultures where there is greater gender inequality (such as Ghana), the issue of honesty in relationships may be more predominant in such societies [40]. Along with the sex differences in the different items in *caring* dimension (reported above), this finding suggests that ‘caring’ as an attribute has a number of different dimensions, with both culture and gender likely to influence their relative importance. Further, this lack of wider systematic differences between egalitarian and more sex-role unequal societies in our data is perhaps less surprising given apparently conflicting recent evidence on societal equality and sex differences in sociosexual orientation and personality traits [13], [41]. This work suggests that, while there may be larger sex differences in human mating strategies in societies in more gender unequal societies, other psychological characteristics (such as personality) may be more similar between the sexes in unequal, rather than sex role egalitarian, cultures. SPMVs are likely to reflect both sexual strategies and broad personality characteristics: further research, ideally with larger numbers of cultures, could profitably examine the inter-relationship between gender role equality and a wider range of relationship attributes, preferences, and strategies.

The present study had several limitations. Our respondents were largely young students from urban areas. Across the lifespan, mate value generally declines for women and increases for men [17], although other factors such as relationship status, fertility, and number of children will influence this [20]. Our SPMV - like our self-esteem [16] - depends on the evaluations of others [20], and we cannot be sure the extent to which these qualities are self-perceived or ‘actually’ possessed by respondents. Indeed, network members are likely to be particularly influential for mate selection in many settings [42]. Although we conducted our pilot work amongst different ethnic groups in the UK our questionnaire was based on lists generated by British respondents. Culturally specific, ‘emic’ items might be particularly relevant in some cultures: In China, for example, filial piety (*Xiao*) is an important factor in mate choice [43], while caste might be more significant in Indian populations. Both the GDI and GEM scores for the White UK samples were taken from national ratings for the United Kingdom, which included all the ethnic minority populations of the UK (e.g. British Asian populations, which comprised around 4% of the UK at the time of study). Finally, we did not specify relationship type when assessing SPMV (e.g. qualities preferred in a husband or wife versus boyfriend or girlfriend). Different qualities may be important when anticipating different ‘relationship futures’ [5]. Similarly, self-esteem is also likely to have different components, each with different adaptive potentials [44]. Future work could usefully examine these in association with SPMV.

Lastly, what are the broader implications of SPMV across cultures? The findings of our study make new contributions to our understanding of our self-perceived mate value, those qualities that make us feel positive about ourselves, and how this varies across sex and cultural context. However, just as particular beliefs might seek to aid or abet such relationships in particular settings [45], particular constellations of self-perceptions might help or hinder relationship development across cultural settings. Some qualities, such as physical attractiveness, may be universally desired and have positive lifelong consequences for not only relationships but other life outcomes [19]. Other characteristics, such as aggressiveness, may be attractive only to moderate degrees [46]. Western research suggests that those high in narcissism may find establishing stable relationships difficult [47], while higher overall SPMV amongst men is related to frequent short term sexual relationships [10]. Future research could profitably further investigate SPMV and relationship stability not only between societies, but intra-culturally, between ethnic and social groups. Such work can then make a further contribution to the largely neglected realm of culture and relationship dynamics.
